# Students’ Awareness of the Role of Phonetics in Construction of Removable Dental Prostheses: A Questionnaire-Based Cross-Sectional Study

**DOI:** 10.3390/dj10120227

**Published:** 2022-12-01

**Authors:** Reem Abualsaud, Ahmad M. Al-Thobity, Masoumah S. Qaw, Danah F. Almaskin, Zahra A. Alzaher, Soban Q. Khan, Mohammed M. Gad

**Affiliations:** 1 Department of Substitutive Dental Sciences, College of Dentistry, Imam Abdulrahman Bin Faisal University, P.O. Box 1982, Dammam 31441, Saudi Arabia; 2 Department of Restorative Dental Sciences, College of Dentistry, Imam Abdulrahman Bin Faisal University, P.O. Box 1982, Dammam 31441, Saudi Arabia; 3 Fellowship Program in Prosthodontics, College of Dentistry, Imam Abdulrahman Bin Faisal University, P.O. Box 1982, Dammam 31441, Saudi Arabia; 4 Department of Dental Education, College of Dentistry, Imam Abdulrahman Bin Faisal University, P.O. Box 1982, Dammam 31441, Saudi Arabia

**Keywords:** removable partial dentures, complete dentures, phonetics, awareness, dental students, dental education

## Abstract

Phonetics plays a major role in the fabrication of prostheses. This study aimed to assess the knowledge of students regarding the role of phonetics in denture fabrication and to improve the educational process and the clinical application. The study was conducted at the College of Dentistry, Imam Abdulrahman Bin Faisal University, and involved a survey of 344 dental students and interns. The questionnaire contained 20 questions and was divided into three sections: general knowledge, clinical correlations, and clinical evaluations. The data were collected and analyzed statistically using independent *t*-tests, one-way ANOVA, and Tukey’s post hoc tests. The response rate was 100%. Male and female students only differed significantly in terms of their scores for answers to general knowledge questions, with females achieving better results (*p* = 0.023). General knowledge varied significantly between fourth-year students and all other levels (*p* < 0.001), and fifth-year students and interns (*p* = 0.027). The clinical correlations varied significantly between fourth-year students and interns (*p* = 0.01), whereas the clinical evaluations varied between all the academic years and interns (fourth-year, *p* < 0.001; fifth-year, *p* = 0.003; and sixth-year, *p* = 0.017). The interns obtained the highest scores in all sections. There was a lack of awareness among dental students of some aspects of the role of phonetics in denture fabrication. The study highlights the deficiencies that need to be addressed and the need for adjustments to the curriculum related to removable prosthodontics in order to improve the knowledge of students regarding the role of speech in denture fabrication.

## 1. Introduction

Awareness of speech allows the dentist to fabricate a prosthesis that meets the goals of oral rehabilitation. Speech is considered one of the most complicated oral activities. It shares many characteristics with mastication, and both constitute oral motor functions [1]. Many factors affect phonation, including mechanical, muscular, aerodynamic, and auditory factors [1]. The tongue plays a key role in speech, mainly through contact with the teeth, lips, alveolar ridge, and the soft and hard palates. In edentulous patients, a prosthesis replaces some of the missing structures, and the dental practitioner needs to understand this phenomenon and have good knowledge to be able to fabricate phonetically suitable dentures [2]. Some anatomical structures that are involved in the production of consonant sounds are relevant to the dentist. The lips produce bilabial sounds, the lips and teeth produce labio-dental sounds, the tongue and teeth produce linguo-dental sounds, and the tongue and hard or soft palate produce lingo-palatal sounds [3].

Patients usually adapt well to speaking with complete dentures, and difficulties that might be encountered when first using a denture should not be permanent or serious complications. The denture should be fabricated so as to fit the patient’s neuromuscular condition rather than require the patient to adapt their abilities to the device [4]. All speech sounds are managed by the air stream that passes through the vocal cords [3,5]. The pharynx, nasal, and oral cavities have valves or articulators that determine the speech sounds [6,7,8]. During speech, the sequences and nature of the sounds are produced through the upward lift of the soft palate to close off the nose [4].

Mechanics, aesthetics, and phonetics are considered the main factors in the production of a successful dental prosthesis [3]. Phonetics determines the alignment and positioning of the upper anterior teeth in complete or partial dentures, which thereby determine the positions of the rest of the teeth [9]. For example, the labio-lingual and inciso-cervical positions of the incisal edges of the maxillary central incisors affect the pronunciation of “F” and “V” sounds [9]. To produce “S” and “Z” sounds correctly, the incisal edges of the mandibular incisors should be slightly lingual to those of the maxillary incisors, with a 1.0–1.5 mm space, which eventually helps to correctly arrange maxillary anterior teeth [10]. Hence, anatomical harmony is achieved together with satisfactory phonetics, lip support, and aesthetics [10]. With regard to the arrangement of posterior teeth, the dorsum of the tongue should have sufficient space to make contact with the maxillary posterior palatal surfaces in the articulation of consonants, such as “D”, “F”, “N”, “K”, “C”, and “S” [11,12].

Vertical dimension and centric relation recordings using various techniques are critical and fundamental steps in denture fabrication [13]. Various methods, including phonetics and speech, need to be used to achieve the proper relationship between the maxilla and mandible. During the try-in appointment, the pronunciation of various words and numbers is assessed, because it is at this stage that the teeth are arranged, and the wax is contoured to mimic the final denture. If the pronunciation of some words or numbers is incorrect, the dentist will modify and correct the positioning of the teeth. For example, when “Mississippi” is pronounced, no contact between the teeth should be detected [14].

With the consonants “T” and “D”, the tongue makes firm contact with the anterior part of the hard palate; this explains why the thickness of the denture base is an important factor in correct phonation [11]. Some other phonetic problems may be detected at the insertion stage. This is because of the differences between the materials used in the last two stages, i.e., the wax and the acrylic. Another reason that problems may be experienced is hypersalivation due to the insertion of the new object in the mouth.

The clinician must be aware of the importance of phonetics when fabricating removable prostheses. Therefore, appropriate knowledge and awareness are required from the beginning of the process. This cross-sectional study aimed to assess the awareness of students concerning the role of phonetics during fabrication of removable prostheses and to help improve the educational process and the clinical application of knowledge. The hypothesis was that students were adequately aware of the role and importance of phonetics in fabrication of complete and partial dentures.

## 2. Materials and Methods

### 2.1. Study Population and Participation

This study was approved by the Institutional Review Board (IRB-2022-02-230) of Imam Abdulrahman bin Faisal University, Dammam, Saudi Arabia. The study was conducted among male and female students who were at the clinical stage of their undergraduate dental studies (fourth–sixth years) in addition to dental interns. The survey was distributed to all dental students (a total of 344) during June and July of 2022, following the delivery of various removable prosthodontic courses. Participation was voluntary and anonymous with the freedom to withdraw at any time. The introductory part of the questionnaire included a statement of agreement to participate which equated to an informed consent. Demographic data, age, sex, and academic year of the participant were recorded in the questionnaire.

### 2.2. Survey Validation and Preparation

The questionnaire was prepared by a prosthodontist and then presented to 3 specialists with more than 15 years of experience in removable prosthodontics. Their opinions regarding the questions and validation of the outcome that each section sought to measure were taken into consideration to finalize the questionnaire. Next, a pilot trial of 10 participants from the target population was conducted to assess the clarity and reliability of the questionnaire. Unclear items were adjusted to eliminate any confusion. The surveys of the participants in the pilot exercise were not included in the study or the statistical analysis.

The questionnaire was a self-administered, paper-based questionnaire in English. It consisted of 20 close-ended questions, including 5 questions aiming to assess the student’s general knowledge about the role of phonetics in the fabrication of removable dentures, eight questions about the clinical correlation of different testing methods with denture fabrication, and 7 questions concerning the clinical steps needed to evaluate trial dentures. Each question had 3 possible answers: correct, incorrect, and I do not know. The answers were coded, and data entry was initially completed using Microsoft Office Excel.

### 2.3. Statistical Analysis

The Statistical Package for Social Sciences (SPSS v.23) was then used for data entry and analysis. Frequency distributions, cross-tabulations, means, and standard deviations were utilized for the descriptive analysis of the data. Each respondent was given a score that was calculated by counting the total correct answers, and a new variable named “Score” was generated. For each section, the minimum score value was 0, and the maximum was the number of questions (i.e., 5 for general knowledge, 8 for clinical correlation, and 7 for clinical evaluation). The normality of the data was tested using the Shapiro–Wilk test and insignificant results proved that the data were normally distributed. Hence, parametric tests were used for the inferential analysis of the data. In inferential statistics, a T-test with two independent samples was used to compare the average score from each section with gender. Furthermore, a comparison between average scores and the academic year of the students was performed using one-way ANOVA followed by Tukey’s post hoc test for pairwise comparisons and *p*-values below 0.05 were considered statistically significant.

## 3. Results

There were 344 students enrolled to complete the questionnaire, and all of them participated in the study (100% response rate). Of these, 161 (46.8%) were males and 183 (53.2%) were females. Table 1 summarizes the questions and responses received for each question in terms of number and percentage. The average general knowledge score was 3.56 (±1.04)/5, the average clinical correlation was 3.2 (±1.44)/8, and the average clinical evaluation score was 3.42 (±1.83)/7. Eleven questions were answered correctly by 50% or more of the participants, whereas nine questions were correctly answered by less than 45% of the participants (Table 1).

Table 2 summarizes the average scores of the three sections of the survey by gender. There was a significant difference between males and females in the general knowledge section with females achieving better results (*p* = 0.023), but not in the clinical correlation (*p* = 0.237) or clinical evaluation (*p* = 0.413) sections.

One-way ANOVA results revealed significant differences between the academic years in the three sections: general knowledge, clinical correlation, and clinical evaluation (*p* < 0.001, *p* = 0.013, and *p* < 0.001, respectively). The average scores in relation to the academic year of the students are set out in Table 3. For general knowledge, the significant variance was between fourth-year students and all other academic years (*p* < 0.001), and between fifth-year students and interns (*p* = 0.027). The performance in the clinical correlation section only varied significantly between fourth-year students and interns (*p* = 0.01). With regard to the clinical evaluation section, there were significant differences between all the academic years and interns (*p* < 0.001, *p* = 0.003, *p* = 0.017), who achieved the highest score in this study (4.2 ± 1.57). In general, the interns achieved higher scores than the other students in all sections. It was notable that scores increased as the students advanced in their undergraduate studies, but as mentioned above, the significance varied.

## 4. Discussion

This study was conducted at Imam Abdulrahman Bin Faisal University and aimed to assess the awareness of students of the role of phonetics during the construction of removable prostheses, in order to help dental institutions provide better educational and clinical guidance. Different academic levels were included to obtain an understanding of the students’ knowledge over the period from the beginning of the clinical year until their graduation. The study results showed variations in the awareness of the students, with multiple values indicating unsatisfactory knowledge of some questions, therefore disproving our study hypothesis.

In the general knowledge questions, there was a significant difference between males and females. The lowest significant score was obtained by fourth-year students. This might be because younger students receive a less detailed and very basic theoretical curriculum compared to older students, in addition to limited clinical exposure which limits their general awareness and experience.

For this section (general knowledge), the lowest scores were evident with the second question, for which 68% of the students chose the incorrect answer. Some pronunciations at the insertion appointment may differ from the try-in stage for multiple reasons; one of these is that the tongue and lips interact differently with wax and processed acrylic resin. Pronunciation with the processed denture is more accurate than with the trial bases [15,16]. This might be the reason why most of the students responded incorrectly, as they have a greater focus on pronunciation when delivering the dentures. However, it is still preferable to assess the phonetics at the try-in appointment [3,17], as the teeth are appropriately arranged and the wax is well contoured. Assessing phonetics at the try-in stage can enable major errors to be recognized and corrected before final denture processing. It is useful to ask the patient to pronounce “Emma”, “Mississippi”, “33”, and “55” at the try-in stage. When pronouncing “Emma” and “Mississippi”, there should be no teeth contact. Saying “33” should provide space between the anterior teeth, whereas the “55” sound is properly produced when the incisal edges of maxillary central incisors contact the wet–dry line of the lower lip without any involvement of the posterior teeth [3,14].

For the same section (general knowledge), the highest score related to the first question, to which 91.6% of the participants gave the right answer: “Correct”. This might be related to the clinical experience of the students, the issues they face when evaluating the phonetics of the patient, and how they correct the mistakes on trial bases. Lip support is essential for correct phonation, and insufficient lip support affects the pronunciation of the letters “B” and “P”, which are produced during contact of the lips [18,19].

Teeth arrangement plays an important role in correct pronunciation. Some sounds are affected by the position of the incisal edge in relation to the lower lip [14,20]. For instance, if the maxillary anterior teeth are positioned too high (more apical), the “V” sound will be pronounced like “F”. In contrast, if the same teeth are positioned more incisally, the “F” will sound more like a “V”. Another example is pronunciation of the letter “S” which needs a close approximation of the maxillary and mandibular incisal edge to allow a slight escape of air [21,22,23]. Additionally, correct phonetics can be used to determine the VDO and ascertain facial height [24]. Any alteration in the VDO can result in pronunciation difficulties [25]. Incorrect VDO can be detected by evaluating the pronunciation of the consonant “S”, for example, by asking the patient to count from 60 to 66 [26].

For the clinical correlation questions, there was a significant difference in the average scores for this section between fourth-year students and interns. This is expected, as the interns are exposed to clinical cases more frequently. In this section, there was no significant difference between males and females.

All but three of the questions in this section were answered correctly by the majority of students. The lowest clinical correlation score was for question 13. Only 28.8% of the students answered this question correctly, whereas the rest either gave an incorrect answer (34%) or did not know the answer (37.2%). “K” and “D” sounds are produced when there is sufficient space for the dorsum of the tongue to contact the palatal surface of the maxillary posterior teeth [3]. Improper faciolingual positioning of maxillary anterior teeth leads to inappropriate pronunciation of “S” and “Z” sounds. These sounds are produced when the incisal thirds of the mandibular anterior teeth are slightly lingual to the incisal third of the maxillary incisors by 1–1.5 mm. The question related to this point (question 11) was answered correctly by 66.3% of participants.

Ninety-seven participants knew that overextension of the posterior border of the denture can be evaluated using the sounds “K”,” G”, and “ng”. These are velar sounds, which are produced by the tongue and hard or soft palate contact. When the denture is overextended, it encroaches on the soft palate and affects the production of these sounds [4]. If patients complain of denture displacement during mouth opening or difficulty swallowing, the dentist can anticipate a problem at the distal extension of the denture. This process can be aided by asking patients to pronounce “K”, ”G”, and “ng”.

Around 42% of the participants did not know the exact function of pronouncing the “Ch” sound. When this sound is produced, the relationship between the anterior teeth can be assessed. The maxillary and mandibular incisors should not touch but approach only in an end-to-end fashion. Therefore, pronunciation of “Ch” does not provide any information on the tooth–tongue relationship or enable the length of the teeth to be assessed. Most of the participants (42.7%) responded “I do not know” to this question. This sound is used to assess the total length of anterior teeth, and their horizontal relationship, in addition to the tongue–alveolus relationship [4,27].

In the clinical evaluation section, there were significant differences between interns and all other students but no difference between males and females. In this section, the lowest and highest scores were obtained by fourth-year students and interns, respectively. This was observed for all the questions and could be attributed to the fact that interns have more knowledge and, most importantly, more clinical experience.

Clinical evaluation questions were answered correctly by the majority of students with the exception of three questions. The lowest correct score (19.2%) was observed for question 17. The words “OLO and ELE” can be used to estimate the vertical dimension. According to Marko et al., 5.5 mm and 7.5 mm can be subtracted from the position of the mandible when the patient says “OLO” and “ELE”, respectively, to obtain the correct vertical dimension [24].

Of the participants, 168 did not know that the “Whistling” sound results when posterior teeth are set too far to the lingual. This may indicate an actual deficiency in knowledge or result from a lack of understanding of the cause of “Whistling”. The Whistling sound results when the anterior tongue is blocked by the upper premolars and, as a consequence, the space becomes too large for the air to escape [3]. In addition, around half of the participants did not know the cause of “Slurry”/s/, /sh/, and /th/ sounds. Here, it is important to note the relationship of the anterior teeth to one other. A poor relationship may cause phonetic problems, in particular when there is inadequate interocclusal space or as a result of the palatal placement of maxillary incisors [3,4].

The best score was for question 16, which was answered correctly by 268 of the participants. This may be related to the exposure of the students to situations requiring the adjustment of the position of anterior teeth. For a patient to pronounce “F” and “V” sounds, the incisal edges of the upper central incisors should contact the lower lip at the moist–dry junction. The difference between “F” and “V” sounds and their relation to teeth positions were mentioned above in the discussion of the first section (general knowledge) [3]. The curriculum of the College of Dentistry has changed in the past few years to introduce clinical practice in earlier years of study, and this might account for the good scores in this section.

This study highlights and confirms the importance of theoretical as well as clinical training for dental students. The authors recommend spending more instructional time or introducing a checklist containing all the mandatory steps for denture fabrication, including phonetics assessment during clinical practice, especially for the early clinical years.

Cross-sectional surveys have been reported to have some limitations [28]. Among the limitations observed in this study is that it was based on a questionnaire. Additionally, the tendency of respondents to provide positive, favorable, socially acceptable [29], or random answers might also be regarded as limitations. Inaccurate responses might result in an overoptimistic or false estimation of the awareness of students. Notwithstanding the study limitations, the outcome of this study provided important clarity regarding the awareness and attitude of dental students towards the role of phonetics in prostheses fabrication, which will ultimately help to improve the educational process. The same study objective could be addressed in future research involving other dental schools, both in the Kingdom and internationally, to improve the awareness and practice of dentists and promote patient care. Such surveys should also be followed up with patient satisfaction and evaluation surveys to ascertain the improvement in the awareness and clinical practice of students.

## 5. Conclusions

The degree of awareness differed according to the level of the student. These differences reflect some deficiencies that require review and standardization of the curricula between different levels. Dental interns performed better in general knowledge, clinical correlation, and clinical evaluation compared to students in all academic years. Fourth-year students displayed a significant deficiency in general knowledge and clinical application. As students advanced in their undergraduate studies, they performed better in understanding and applying phonetics in complete dentures. This investigation suggests a need for a review of the curriculum and the addition of specific lectures regarding the importance and function of phonetics during prosthesis fabrication, in addition to the formulation of a checklist for all denture fabrication steps and assessments. This would result in a better understanding of the concepts and the provision of better treatment for improved patient satisfaction.

## Figures and Tables

**Table 1:** Proportion of correct/incorrect/I do not know responses for each question. Bold font indicates the correct answer.

General Knowledge	n (%)		
	Correct	Incorrect	I Do Not Know
Improper pronunciation maybe the result of unsupported lips, errors in teeth positioning, or altered facial height.	**315 (91.5)**	14 (4.1)	15 (4.3)
2. Phonetics evaluation is preferably performed at insertion appointment.	234 (68.0)	**94 (27.3)**	16 (4.7)
3. Major phonetic problems could result from increased vertical dimension of occlusion (VDO).	**266 (77.3)**	34 (9.9)	44 (12.8)
4. Phonetic method is the most popular method for vertical dimension of rest (VDR) recording.	**108 (31.4)**	112 (32.6)	124 (36.0)
5. Teeth position and proper denture base thickness are important for adequate speech.	**303 (88.1)**	20 (5.8)	21 (6.1)
**Clinical correlation**			
6. “S” and “SH” are sounds that are created by air alone. Therefore, they are called voiceless speech sounds.	**144 (41.9)**	113 (32.8)	87 (25.3)
7. “F” and “V” sounds are formed between the lips (Bilabial).	105 (30.5)	**200 (58.1)**	39 (11.4)
8. “T”, “D” and “N” sounds appear when the tongue is pressed firmly against the anterior hard palate (tongue and hard palate).	**278 (80.8)**	27 (7.8)	39 (11.4)
9. “TH” sound is used to assess the height of maxillary incisors.	34 (9.9)	**218 (63.4)**	92 (26.7)
10. “CH” sound is used to assess the length of maxillary incisors and tongue-tooth relation.	97 (28.2)	**100 (29.1)**	147 (42.7)
11. The incisal edges should approximate each other, but not touch during the pronunciation of “S” sound.	**228 (66.3)**	62 (18)	54 (15.7)
12. Overextension of posterior (distal) border of maxillary denture may be evaluated using some sounds.	**97 (28.2)**	126 (36.6)	121 (35.2)
13. Incorrect horizontal and vertical positioning of maxillary incisors leads to mispronunciation of “K”, and “D” sounds.	117 (34)	**99 (28.8)**	128 (37.2)
**Clinical evaluation**			
14. To evaluate proper vertical dimension, ask the patient to say “M”.	**221 (64.2)**	59 (17.2)	64 (18.6)
15. To check the height of anterior part of upper wax rim, ask the patient to say “S”.	**179 (52.0)**	60 (17.5)	105 (30.5)
16. At try-in appointment, when the patient pronounces the letter “F”, the lower lip should be in light contact with the incisal edges of maxillary incisors.	**268 (77.9)**	30 (8.7)	46 (13.4)
17. “OLO” and “ELE” are used for vertical dimension (VD) evaluation.	**66 (19.2)**	42 (12.2)	236 (68.6)
18. “Whistling” sound results when posterior teeth are set too far lingually.	**102 (29.7)**	74 (21.5)	168 (48.8)
19. “Slurry” /s/, /sh/, and /th/ sounds are caused by palatal placement of maxillary incisors or excess denture base thickness palatal to them.	**155 (45.1)**	28 (8.1)	161 (46.8)
20. “F” sound will turn out like “V” sound if upper anterior teeth are arranged below the occlusal plane.	**186 (54.1)**	39 (11.3)	119 (34.6)

**Table 2:** Average score for each section by gender.

Gender	Mean(SD)		
	General Knowledge	Clinical Correlation	Clinical Evaluation
Male	3.42(1.1)	3.1(1.5)	3.34(1.9)
Female	3.7(0.97)	3.3(1.4)	3.5(1.7)
*p*-value	0.023 *	0.237	0.413

* statistically significant at 0.05 level of significance.

**Table 3:** Average score for each section in relation to academic year with results of Tukey’s post hoc test.

Level	Mean(SD)		
	General Knowledge	Clinical Correlation	Clinical Evaluation
Fourth	2.9(1.1)	2.8(1.4) ^a,b^	2.7(2.1) ^a,b^
Fifth	3.5(0.99) ^a^	3.1(1.39) ^a,c,d^	3.3(1.7) ^a,c^
Sixth	3.7(1.0) ^a,b^	3.3(1.47) ^b,c,e^	3.4(1.71) ^b,c^
Interns	3.9(0.83) ^b^	3.5(1.41) ^d,e^	4.2(1.57)

The same letters in each column indicate statistically insignificant differences between the pairs.

## Data Availability

Not applicable.
